# Occurrence, spatiotemporal distribution, and ecological risks of antibiotics in a river basin of eastern China

**DOI:** 10.1007/s10661-026-16036-z

**Published:** 2026-10-16

**Authors:** Xintong Lin, Jing Wang, Zhengzheng Liu, Jingqing Zhou, Qinqin Sun, Shuxin Chen, Huayu Hu

**Affiliations:** 1 School of Civil and Environmental Engineering, The University of New South Wales, Sydney, NSW 2052 Australia; 2 Ecological and Environmental Monitoring Center of Zhejiang Province, Hangzhou, 310012 China

**Keywords:** Surface water, Pollution characteristics, Source identification, Ecological risk assessment, Seasonal variation

## Abstract

The widespread use of antibiotics has resulted in their frequent occurrence in aquatic environments, raising ecological concerns; however, conducting precise monitoring and tracing of a broad spectrum of antibiotics concurrently in complex river networks at the watershed scale still faces significant challenges. This study investigated the pollution characteristics of 32 target antibiotics in a major river basin in eastern China. A total of 184 river water samples collected across 2024–2025 were analyzed. Both detection frequencies (DFs, 0–82.86%) and concentrations (0–36,577 ng/L) of the antibiotics exhibited pronounced spatiotemporal and compound-specific variations. The risk quotient (RQ), calculated as the ratio of the detected concentration to the predicted no-effect concentration, ranged from 0 to 18.92. Sulfachloropyridazine (SCP), sulfamethoxazole (SMZ), and sulfathiazole (STZ) were identified as priority antibiotics for routine monitoring due to high DFs (≥ 50%) or high RQs (> 1 in some sampling points). In contrast, 14 antibiotics that were not detected in any samples and 10 others that exhibited consistently low DFs (< 20%) and low RQs (< 0.1) were assigned lower monitoring priority. Spatial analysis revealed two independent pollution hotspots dominated by SCP and by SMZ and florfenicol, likely sourced from livestock farming and urban sewage, respectively. The apparent attenuation coefficient of SCP, derived from the field monitoring data, revealed both seasonal and spatial variations, ranging from 0.28 to 2.24 km^−1^. These findings provide a high-resolution spatiotemporal dataset and identify key antibiotics for future monitoring and targeted management in similar river systems.

**Supplementary Information:**

The online version contains supplementary material available at https://doi.org/10.1007/s10661-026-16036-z.

## Introduction

With the rapid advancement of global industrialization and urbanization, the emission and accumulation of new organic pollutants in water bodies have become a significant environmental issue worldwide (Tran et al., 2018). Among them, pharmaceuticals and personal care products, especially antibiotics, are regarded as an important group of new pollutants due to their continuous input, environmental persistence, biological activity, and potential ecological toxicity (Kulik et al., 2023; Petrie et al., 2015). Studies have shown that even if the concentrations of antibiotics detected in surface water are below clinical levels, they can still have toxic effects on aquatic organisms (especially single-celled prokaryotes and eukaryotes), thus requiring an ecological risk assessment (Danner et al., 2019; Kulik et al., 2023). Firstly, they directly cause ecological toxic effects, with primary producers such as algae and cyanobacteria being particularly vulnerable, potentially disrupting the basic trophic levels and ecosystem functions (Li et al., 2022). Secondly, what might have more profound and long-term consequences is that they act as selective stressors, driving the proliferation and environmental dissemination of antibiotic resistance genes and antibiotic-resistant bacteria. This weakens the effectiveness of important clinical treatment methods and poses a profound and escalating threat to global public health security, making the monitoring of antibiotic levels in surface water not only an ecological issue but also a pressing biomedical imperative (Pruden et al., 2006).

The monitoring work conducted over the past two decades has revealed the widespread presence of antibiotics in diverse aquatic environments globally, including rivers, lakes, estuaries, and interconnected groundwater systems (Coderre et al., 2025; Hughes et al., 2013; Wu et al., 2022). Antibiotics from various classes such as sulfonamides (SAs), fluoroquinolones (FQs), macrolides (MCs), and tetracyclines (TCs) are routinely detected across climatic regions, from humid basins to arid oasis systems, with concentrations typically spanning the ng/L to µg/L range (Anh et al., 2021; Danner et al., 2019; Wu et al., 2022). This pervasive contamination underscores the transboundary nature of the issue, and the problem is particularly severe in developing countries, densely populated areas, and surrounding river basins of livestock farming areas (Adedipe et al., 2024; Coderre et al., 2025). For instance, the Asian river systems are severely polluted, and antibiotics such as sulfamethoxazole, norfloxacin (NOR), and erythromycin (ERY) are frequently detected at relatively high concentrations (Anh et al., 2021; Li et al., 2018). In the Fenhe River Basin of northern China, 20 antibiotics were recently detected, with SAs showing the highest average concentration (118.30 ng/L); NOR, ofloxacin (OFX), and ERY were identified as priority ecological risk pollutants, with peak concentrations reaching approximately 2.4-, two-, and ninefold of their respective risk thresholds (Liu et al., 2025). Similarly, the Latin American basins (such as the Iguazu River in Brazil) have also been documented worrying concentrations in the µg/L range, associated with the lack of wastewater treatment facilities (Coderre et al., 2025; Loos et al., 2013).

There are significant differences in the environmental input pathways of different types of antibiotics, which enables the pollution spectra of antibiotics to serve as a “fingerprint” for tracing the pollution sources to a certain extent (Becker et al., 2016). Sulfonamides and tetracyclines are usually closely related to the fecal emissions from livestock and aquaculture industries, and their concentrations are abnormally high in downstream water bodies in the breeding areas (Liu et al., 2024; Lulijwa et al., 2020). Macrolides (such as ERY and azithromycin) and fluoroquinolones (such as OFX and ciprofloxacin) are more likely to originate from human medical activities and enter the environment through domestic sewage and hospital wastewater, resulting in their prevalence in densely populated urban areas and downstream of sewage treatment plants (Coderre et al., 2025; Li et al., 2022). Specific drugs, such as lincomycin, have been identified as characteristic pollutants related to urban sewage in certain river basins (Anh et al., 2021). Therefore, analyzing the composition characteristics of antibiotics in surface waters enables effective distinction and quantification of pollution contributions from different human activities. Similarly, identifying land use types can help predict the relative proportion of various antibiotics in receiving water bodies, thereby facilitating the preliminary identification of antibiotic markers for a given river basin.

Despite the continuous deepening of understanding, conducting precise monitoring and tracing of antibiotics in complex river networks and water systems still faces significant challenges. Various antibiotics coexist in the environment and their sources are intertwined, making it difficult to clearly identify the main contributors to pollution solely based on concentration data. Meanwhile, although the correspondence between antibiotic types and macroscopic pollution sources (such as aquaculture and sewage) has been preliminarily established, how to select specific characteristic antibiotics that are applicable to a particular river basin and can be used for precise tracing still requires local verification based on the actual pollution gradient. Many existing studies either have limited coverage of target antibiotics or insufficient spatial resolution, making it difficult to systematically depict the complete migration path of pollutants from upstream to downstream, throughout urban and rural areas and agricultural zones. Therefore, it is still needed to conduct systematic research at the watershed scale.

Specifically, while extensive monitoring studies have documented antibiotic occurrence in Chinese river systems (Li et al., 2018; Liu et al., 2024), critical knowledge gaps persist. Most previous studies at the watershed scale either only reported the distribution patterns of pollutants without linking them to the attenuation dynamics of specific pollution sources or relied solely on qualitative inferences about pollution sources without quantitative verification. Moreover, existing antibiotic monitoring priority frameworks often adopt general standards and fail to adapt to the unique pollution characteristics or spatial heterogeneity of different watersheds. To address these deficiencies, we selected representative sections along the mainstream, main tributaries, and drinking water source areas of a large river basin in eastern China, and conducted systematic analyses for 32 typical antibiotics including 10 SAs, 11 FQs, 3 TCs, 4 MCs, 2 lincosamides (LINs), and 2 amphenicols (AMPs) across a 2-year span. By analyzing the occurrence levels and spatiotemporal distribution characteristics of the 32 antibiotics in the river water, we aimed to (1) elucidate the spatiotemporal distribution patterns and pollution hotspots of antibiotics within the basin and (2) assess the ecological risks posed by detected antibiotics to aquatic ecosystems and identify the key risk indicators. In addition, after combining the antibiotic compositional profiles with land use data, potential pollution sources and attenuation coefficients of identified antibiotic markers were analyzed. The findings are expected to provide a scientific basis for establishing an efficient monitoring program and implementing targeted pollution control strategies in this and similar river basins.

## Materials and methods

### Chemicals and reagents

The detailed information of the 32 antibiotics is shown in Table 1 and Table S1 in the Supporting information. All standard antibiotics were purchased from Shanghai Yuanye Biotechnology Co., Ltd., China, with a purity of over 98% (TYL purity over 93%). Methanol was purchased from Fisher Chemical (USA), ammonia water was purchased from TCI (USA), the enrichment packing column (HLB) was purchased from Waters Oasis, and 0.45 µm water-based filter membrane was from Whatman (UK). The purity of all reagents used was mass spectrometry grade. Ultrapure water was used from a Milli-Q system.

**Table 1:** List of target antibiotics with corresponding abbreviations, concentration ranges, and detection frequencies (DFs) in the studied river basin

Antibiotics		Abbr.	Conc. range (ng/L)	DFs	
				DF (%)	Categories
Sulfonamides (SAs)	Sulfachloropyridazine	SCP	0.10–36600	57.1	High detection
	Sulfamethoxazole	SMZ	0.05–77.1	56.5	High detection
	Sulfameter	SMD	0.10–3090	30.4	Medium detection
	Sulfathiazole	STZ	0.05–831	26.6	Medium detection
	Sulfamethazine	SM2	0.05–32.4	4.35	Low detection
	Sulfapyridine	SPD	0.05–19.5	3.80	Low detection
	Sulfadiazine	SDI	0.05–4.32	0.54	Low detection
	Sulfamerazine	SM	0.10	0	No detection
	Sulfamonomethoxine	SMM	0.05	0	No detection
	Sulfamethizole	SMTZ	0.10	0	No detection
Fluoroquinolones (FQs)	Ofloxacin	OFX	0.25–46.0	6.52	Low detection
	Norfloxacin	NOR	0.30–10.9	1.09	Low detection
	Sparfloxacin	SPX	0.25–1.83	0.54	Low detection
	Enrofloxacin	ENR	0.50	0	No detection
	Ciprofloxacin	CP	0.25	0	No detection
	Sarafloxacin	SAR	0.25	0	No detection
	Enoxacin	ENX	0.25	0	No detection
	Lomefloxacin	LMFX	0.55	0	No detection
	Nalidixic acid	NA	0.20	0	No detection
	Flumequine	FLU	0.10	0	No detection
	Cinoxacin	CIN	0.10	0	No detection
Tetracyclines (TCs)	Oxytetracycline	OTC	0.25	0	No detection
	Tetracycline	TC	0.50	0	No detection
	Chlortetracycline	CTC	0.50	0	No detection
Macrolides (MCs)	Azithromycin	AZM	0.45–46.7	26.1	Medium detection
	Erythromycin	ERY	1.25–6.89	19.0	Low detection
	Clarithromycin	CLR	0.45–11.0	19.0	Low detection
	Roxithromycin	ROX	0.50–12.5	1.63	Low detection
Lincosamides (LINs)	Lincomycin	LIN	0.05–44.7	46.2	Medium detection
	Clindamycin	CLI	0.05–9.81	37.0	Medium detection
Amphenicols (AMPs)	Florfenicol	FF	0.05–70.9	26.6	Medium detection
	Chloramphenicol	CAP	0.10–13.1	2.17	Low detection

The minimum concentrations of all antibiotics equal to half of their LOD values

### Water sample collection

**Fig. 1:**
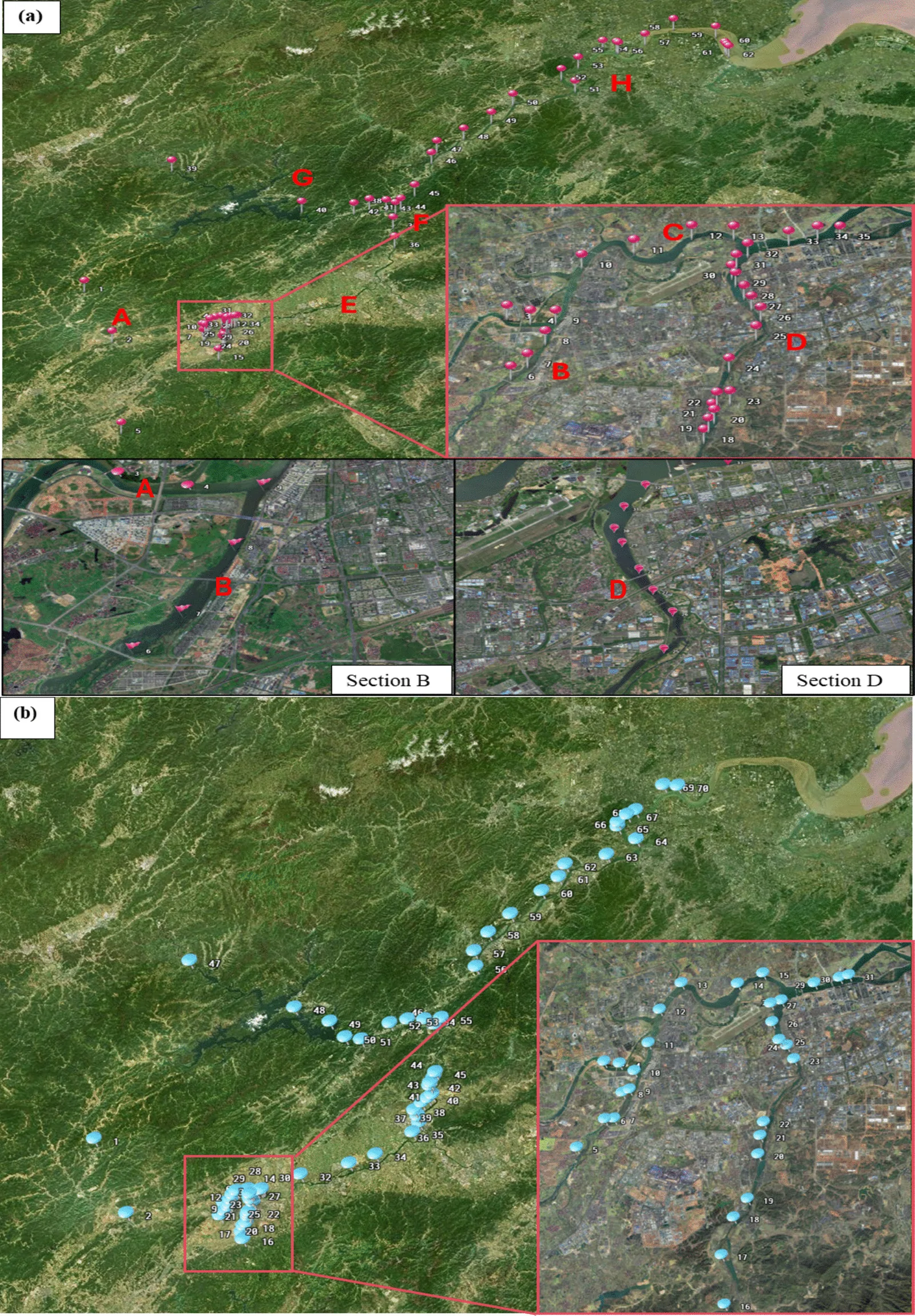
The maps showing sampling point distributions in **a** 2024 (May, *n* = 62; October, *n* = 52, overlapping the May points) and **b** 2025 (October, *n* = 70). The magnified views in panel show the land use types of specific river sections B and D. The letters A to H stand for the eight river sections

The selected river basin is a complete river network system formed by several major streams and their tributaries (Fig. 1). The mainstream flows approximately 668 km downstream through extensive river valleys before entering the East China Sea, with the entire drainage basin of about 55,558 km^2^. The upstream and source areas of major tributaries are mainly characterized by natural ecological land uses such as forests and grasslands, which are important water source conservation areas and ecological barriers for the basin. In the hilly and river valley plain areas in the middle reaches, the land use is mainly composed of cultivated land (paddy fields and dry land), which is a traditional area for grain and specialty economic crop production. However, there are also scattered industrial parks and urban construction lands. In the plain and delta areas downstream, land use is highly intensive, with dense urban construction land, industrial and transportation land, and agricultural land mainly featuring high-yield and efficient precision agriculture and suburban agriculture. At the estuary area, there coexist tidal wetland ecosystems and high-intensity port and port-related industrial land. Overall, the land used in the river basin is deeply influenced by human activities. From the upstream to the downstream, the proportion of natural and semi-natural landscapes decreases, while the proportion of artificial landscapes increases, reflecting the typical land use transformation trajectory of a composite river basin under rapid economic and social development.

A total of 184 water samples were collected across May 2024, October 2024, and October 2025, with the sampling positions shown in Fig. 1. In May 2024, there were 62 sampling points, 52 of which were conducted the second round of sampling in October 2024; therefore, when comparing annual concentration variations, the average of the two measurements from the repeated sampling sites in 2024 was used. In 2025, samples were collected from 70 sites, mainly filling the gaps in sampling points in river sections E and F (Fig. 1), providing a more complete database. Four liters of surface water at each sampling point were collected with the surface water sampler and stored in brown glass bottles. The samples were then kept at 4 ℃ before analyses.

### Water sample pretreatment and analyses

The sample pretreatment and analysis methods refer to our previous study (Chen et al., 2020). Briefly, the collected water samples undergo solid-phase extraction (SPE) using SPE Automatic Solid Phase Extraction Instrument (ZQ31003). Oasis HLB packing column was selected as the enrichment packing column due to its excellent hydrophilic and lipophilic properties, following the procedures below. Five milliliters methanol and 5 mL ultrapure water were used to activate the HLB column; 500 mL water sample with pH adjusted to 5 passed through the packing column at a flow rate of 5 mL min^−1^; 5 mL ultrapure water was used to wash the packing column, followed by nitrogen blow drying for 15 min; 5 mL of methanol was used to soak the packing column and elute; 5 mL of 5% NH_4_OH methanol solution was used to elute the packing column. The enriched extract was evaporated under a gentle nitrogen stream at 45 ℃ to a final volume of 1 mL, and then transferred to vials for instrumental analysis. To ensure quality control, each batch involved 30 samples, including 2 quality control samples (1 full procedure blank and 1 solvent blank) and 28 water samples. No antibiotics were detected in the procedure blanks and solvent blanks.

Ultra-performance liquid chromatography tandem mass spectrometry (UPLC-MS/MS, QTRAP 5500) was used to analyze the antibiotics. C18 column was used as the stationary phase, maintaining at 40 ℃. Formic acid aqueous solution (0.1%) was used as mobile phase A, while methanol solution was used as mobile phase B. The elution gradient ratio of the mobile phase is shown in Table S2. The flow rate was kept at 0.3 mL min^−1^. In the mass spectrometry analysis, the positive ion electrospray ionization mode (ESI +) was employed to ionize the antibiotics, and the multiple reaction monitoring (MRM) mode was used for simultaneous monitoring of the antibiotics. Other parameters were set as follows. The blowing curtain pressure was 20 Psi, the atomization temperature was 550 ℃, both the atomization gas (Gas 1) and the auxiliary gas (Gas 2) were 55 Psi, and the spray voltage was 5500 V.

External standard method was used to quantify the pollutant concentration. The standard curve was set with 7 concentration gradients, from 1 to 200 µg/L. Meanwhile, the internal standard was used to calibrate the standard curve. The limits of detection (LOD) and quantification (LOQ) of the antibiotics were 0.1–2.5 ng/L and 0.4–10 ng/L, as listed in Table 1 and Table S1, respectively.

### Environmental risk assessment

Risk quotient (RQ) that has been widely used to evaluate the risk of drugs in water bodies (Zainab et al., 2020) was employed for the ecological risk assessment of antibiotics. RQ can be calculated according to Eq. (1) (Ågerstrand et al., 2015; U.S. Environmental Protection Agency [EPA], 2022a; Liu et al., 2024).

1 $$\mathrm{RQ}=\mathrm{MEC}/\mathrm{PNEC}$$

where MEC represents the measured concentration (ng/L); PNEC represents the predicted no-effect concentration (ng/L), as calculated by Eq. (2) (Qin et al., 2020).

2 $$\mathrm{PNEC}={\mathrm{LC}}_{50}/\mathrm{AF}$$

where LC_50_ represents the median lethal concentration (ng/L), which is calculated using the ECOSA model (European Commission [EC], 2003; EPA, 2022b), as summarized in Table S3; AF is the evaluation factor, which is set at the recommended value of the EU Water Framework Directive (AF = 1000) (Hernando et al., 2006; ECHA, 2016). Research results have shown that an RQ of < 0.01 indicates no risk, 0.01 ≤ RQ < 0.1 represents a low risk, 0.1 ≤ RQ < 1 indicates a medium risk, and RQ ≥ 1 represents a high risk (Hernando et al., 2006; Wang et al., 2023; Zainab et al., 2020).

### Attenuation coefficient calculation

A graphical analysis method based on logarithmic linearization of concentration was adopted to determine the apparent attenuation coefficient. The analysis was conducted using data from river section B (sampling points 6–9 in 2024 and points 7–10 in 2025), where sulfachloropyridazine (SCP) exhibited a clear isolated concentration peak followed by a downstream declining trend without major tributary confluences or suspected point sources in between. This selection minimizes the confounding effects of additional input, allowing the estimation of in-stream attenuation. The monitoring point data in the high-value area were taken as the natural logarithm to obtain logarithm of the concentration (ln C). Software Origin 2017 was used to draw a distance–ln C scatter plot. The data points showed a clear linear distribution trend, indicating that the concentration attenuation conforms to the first-order kinetic model: *C* = *C*₀ e^–*λx*^, where λ is the attenuation coefficient based on distance (km⁻^1^), equal to the negative slope of the regression line, *x* is the distance between the peak value (*C*₀) point and its downstream point, and *C* is the concentration of the target antibiotic at the downstream point. It should be noted that this coefficient represents an aggregate field-scale estimate integrating dilution, hydrodynamic mixing, sorption, photolysis, and biodegradation, rather than an intrinsic degradation rate constant. The estimated λ values should therefore be interpreted as apparent attenuation coefficients specific to the field conditions of this river section.

### Principal component analysis (PCA)

To identify the underlying patterns of antibiotic contamination and explore the complex relationships among different antibiotic groups across various sampling scenarios, PCA was performed. First, due to the significant variability in antibiotic concentrations, all datasets underwent a base-10 logarithmic transformation (log10) to reduce the skewness caused by high-concentration outliers. Subsequently, the log-transformed data were standardized using Z-score normalization to eliminate the dimensional differences between various antibiotic parameters. The PCA was conducted on the standardized matrix to extract principal components (PCs) that capture the maximum variance within the dataset. The score plot was utilized to visualize the clustering of samples based on seasonal and spatial variations, while the loading plot was used to interpret the contribution of individual antibiotics to the principal components, thereby identifying the key drivers of water quality variation. These two plots are generated using MATLAB (R2026a).

### Quality control and statistical analysis

Field duplicate samples were collected at approximately 10% of the sampling sites to evaluate sampling precision. All samples were analyzed in batches, each including a procedural blank and a solvent blank to monitor potential contamination during the analysis. Instrument performance was verified daily using calibration standards. The relative standard deviations (RSDs) for duplicate samples were less than 15% for all target compounds.

## Results and discussion

### Detection frequencies of antibiotics

The detection frequencies (DFs) of 32 antibiotics vary significantly, depending on the sampling time, location, and antibiotic type (Figure S2). Following similar approaches in the literature (Anh et al., 2021; Liu et al., 2022), we categorized DFs into four levels: no detection (0%), low detection (0–20%), medium detection (20–50%), and high detection (> 50%). A total of 14 antibiotics were not detected across the three sampling times in 2024 and 2025, which were SM, SMM, SMTZ, ENR, CP, SAR, ENX, LMFX, FLU, NA, CIN, OTC, TC, and CTC; 10 antibiotics were categorized into the low detection level, including SM2 (4.35%), SPD (3.80%), SDI (0.54%), OFX (6.52%), NOR (1.09%), SPX (0.54%), ERY (19.02%), CLR (19.02%), ROX (1.63%), and CAP (2.17%); 6 antibiotics were included into the medium detection level, being SMD (30.43%), STZ (26.63%), AZM (26.09%), LIN (46.20%), CLI (36.96%), and FF (26.63%); SCP (57.07%) and SMZ (56.52%) were categorized into the high detection level.

The 14 undetected and the 10 with low DFs could have minimal impact on the river basin, and therefore, their monitoring frequencies could be reduced in future surveillance programs. However, given that environmental occurrence can shift with changes in agricultural practices, consumption patterns, or hydrology, a periodic reassessment is recommended to verify that these compounds remain at negligible levels. Typical antibiotics from livestock operations, such as tetracyclines (OTC, TC, CTC) and enrofloxacin (ENR) were not detected, suggesting that livestock wastewater may not be the primary pollution source in this basin, contrasting with findings from intensive livestock farming regions worldwide (Sarmah et al., 2006; Kemper, 2008). The high DFs of SCP and SMZ make them strong candidate marker compounds for antibiotic monitoring in this river basin. Existing research has also identified SAs as a key risk category, with SMZ being frequently detected in surface waters globally (Anh et al., 2021; Coderre et al., 2025; Liu et al., 2024).

### Spatiotemporal distribution and source identification

**Fig. 2:**
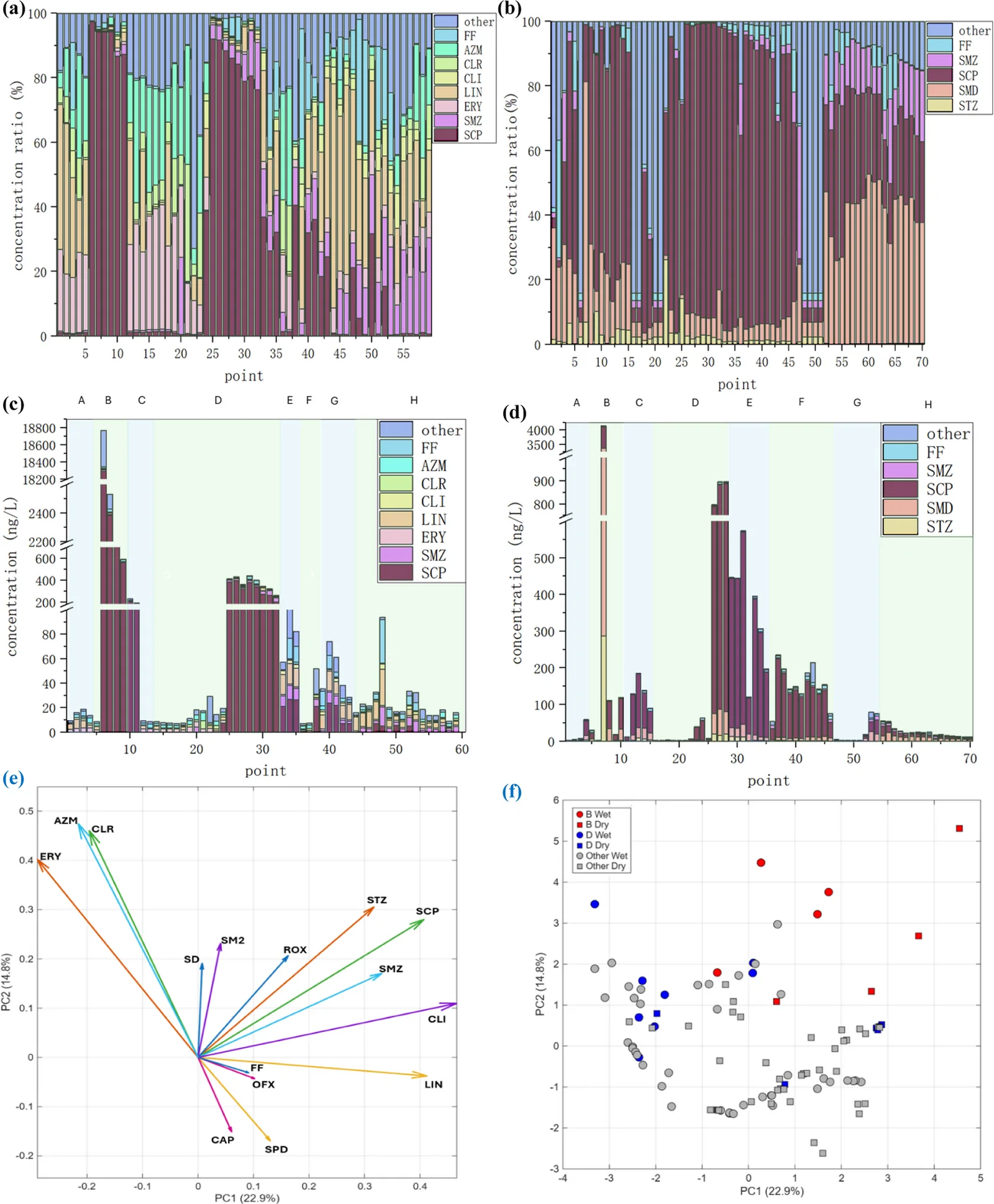
The concentrations (**a**, **c**) of antibiotics and their ratios (**b**, **d**) at each sampling point in 2024 (**a**, **b**) and **2025** (**c**, **d**). **e** Principal component analysis (PCA) loading plot showing the contributions and directions of individual antibiotics to PC1 and PC2. **f** PCA score plot showing sample distribution along the first two principal components (PC1 and PC2). Abbreviations: AZM, azithromycin; CLR, clarithromycin; ERY, erythromycin; STZ, sulfathiazole; SCP, sulfachloropyridazine; SM2, sulfamethazine; ROX, roxithromycin; SMZ, sulfamethoxazole; CLI, clindamycin; FF, florfenicol; OFX, ofloxacin; CAP, chloramphenicol; SPD, sulfapyridine; LIN, lincomycin; SMD, sulfameter; SDI, sulfadiazine

Figure 2 provides an overview of the spatial distribution of detected antibiotics in the river basin. From an overall perspective, the total antibiotic concentrations at each sampling point varied significantly, indicating that the pollution input was not uniformly distributed but rather had obvious local hotspots. Among the compounds detected, SCP occupied an absolutely dominant position, with the annual average concentration up to 18,288 ng/L in the river section B, a level significantly exceeding typical surface water concentrations reported globally (generally < 1000 ng/L; Liu et al., 2024). In addition, the graphs showing the concentration ratios (Fig. 2a, b), SMZ and FF also accounted for considerable proportions in both sampling years. In contrast, LIN, ERY, and AZM that had considerable proportions in 2024 were hardly detected in 2025.

The spatial distribution of antibiotic concentrations showed significant discontinuity and spatial heterogeneity. In 2024 (Fig. 2c), two spatially independent pollution high-value zones were observed. The first high-value zone (river section B) was located at a major tributary upstream of the river basin, exhibiting extremely high total antibiotic concentrations that were dominated by SCP. This area presented typical “high intensity, rapid attenuation” characteristics, with concentrations decreasing sharply downstream (points 6–9). The abnormal high concentration at point 6 strongly suggests a powerful and localized sulfonamide source, whose intensity was sufficient to overshadow the contributions of all other antibiotics and unable to be explained by dispersed domestic sewage. The second high-value zone (river section D, points 25–32) was dominated by SMZ and FF. Except for the sampling point 25 that had a relatively low concentration, the remaining sites were consistently high. This zone exhibited “medium intensity, slow attenuation” characteristics, with concentrations declining gradually along the flow path. The two high-value zones were separated by a river section with background concentration levels (below 50 ng/L), indicating that the two tributaries could have distinct pollution sources with different emission intensities and in-stream environmental behaviors. A similar spatial pattern was observed in 2025 (Fig. 2d), though with lowered concentrations in river section B and elevated concentrations in river section D.

PCA of the 15 detected antibiotics separated SCP-dominated samples from AZM/ERY-dominated samples along PC2, reflecting the dominant influence of SCP’s extreme concentrations on the overall dataset. Notably, SMZ and FF—the dominant compounds in the D-section hotspot—did not load strongly on PC1 or PC2, suggesting that their occurrence was less tightly coupled to the primary source gradients captured by PCA. This limitation likely arises from the relatively low concentrations of SMZ and FF (max. 77.1 and 70.9 ng/L, respectively) compared to SCP (max. 36,577 ng/L), which dominates the variance structure of the dataset.

SCP is a widely used SAs in the veterinary field, especially in the livestock industry (Sarmah et al., 2006; Zainab et al., 2020). Based on the Esri Land Cover land use type map (Figure S1), the sampling point 6 located downstream of an intensive livestock farm, where SCP, as a possible feed additive or therapeutic drug in the breeding process, could be directly or indirectly discharged into the river through the surface runoff from the breeding wastewater or manure returned to the fields. However, this represents a plausible source identification based on spatial coincidence and compound-specific signatures, rather than a quantitatively demonstrated source apportionment. Receptor models (e.g., positive matrix factorization or absolute principal component score-multiple linear regression) or stable isotope analysis would be required for confirmatory source apportionment. Despite this limitation, the observed spatial pattern provides strong circumstantial evidence that a localized agricultural source is the most parsimonious explanation. This case profoundly illustrates that even in the context of large-scale environmental quality improvement, point-source or concentrated non-point source pollution driven by specific land use types (such as intensive agriculture) remains the main cause of local high risks. Future watershed management needs to implement more targeted control measures for such “key source areas.”

### Comprehensive analysis of SCP in-stream attenuation

**Fig. 3:**
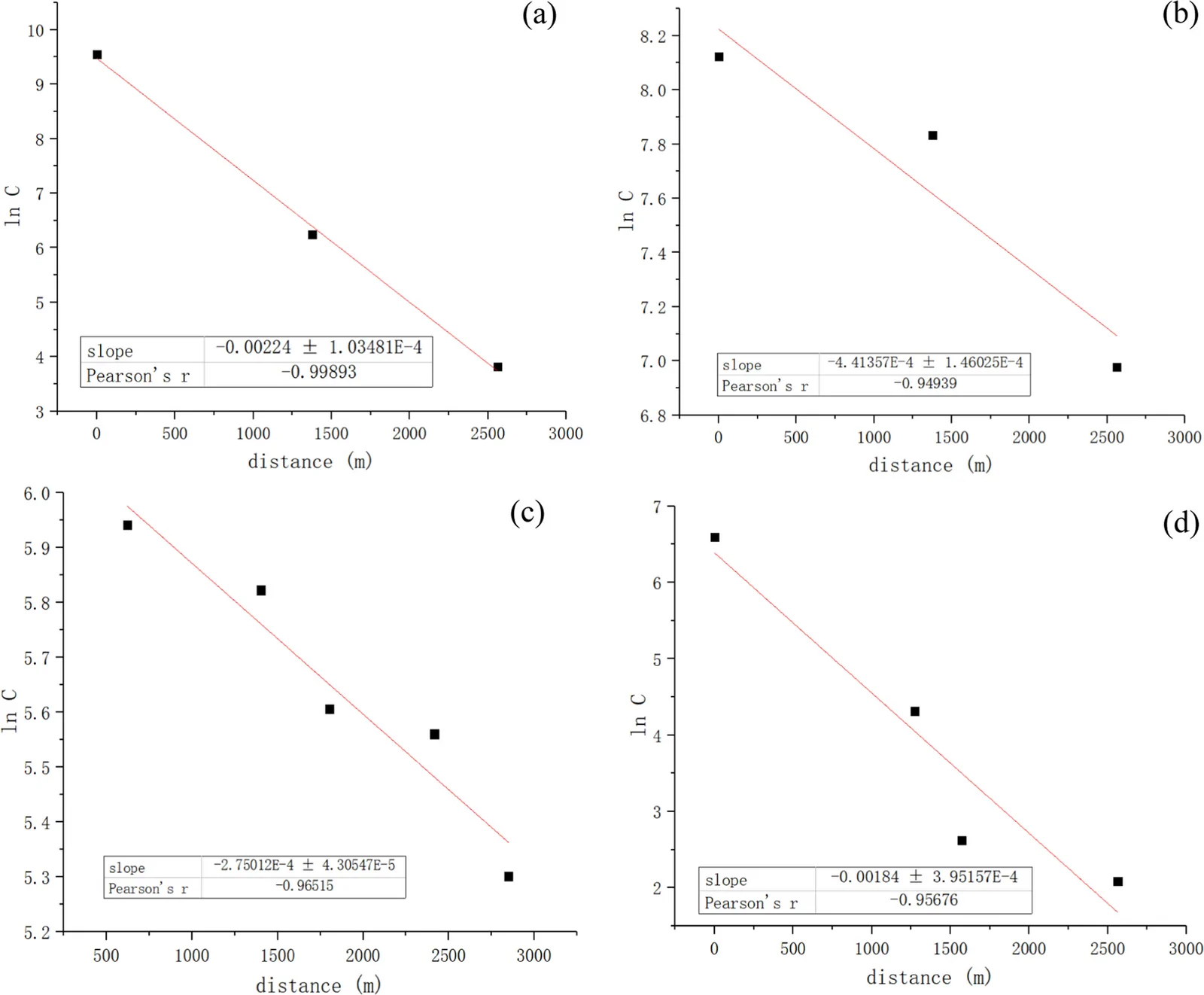
Apparent field attenuation coefficients (λ, km⁻^1^) of sulfachloropyridazine (SCP) derived from linear regression of ln concentration versus downstream distance. **a** River section B, May 2024 (wet season); **b** River section B, October 2024 (dry season); **c** River Section D, 2024 (Average); **d** River section B, 2025

Given the “high-intensity, rapid-attenuation” pattern of SCP pollution, understanding its natural attenuation is imperative for assessing its persistence and informing targeted regulation. However, most existing researches have not considered SCP as a focus antibiotic. On February 25, a total of 13,445 results were retrieved from the Web of Science Core Collection by searching the topic field using the terms “antibiotics” AND “surface water,” while substituting “antibiotics” with “sulfachloropyridazine” returned only 48 publications. This research gap can be likely due to the rapid degradation characteristics of SCP in the environment, which poses a challenge for its accurate quantification. In view of this, a simple linear regression analysis was conducted on the obtained data from this study to estimate the attenuation rate (*λ*) of SCP (Fig. 3). For river section B, *λ* was 2.24 km⁻^1^ (95% confidence interval (CI) 0.93–3.55 km⁻^1^) in the 2024 wet season, a comparable but higher value than that observed in the 2025 wet season (1.84 km⁻^1^, 95% CI including zero). However, a substantial decrease to 0.44 km⁻^1^ (95% CI including zero) was observed in the 2024 dry season, which was largely comparable to the obtained *λ* (0.28 km⁻^1^, 95% CI 0.14–0.41 km⁻^1^) in the same season for river section D (points 27–32). The relatively high coefficients of determination (*R*^*2*^ = 0.998, 0.901, 0.932, and 0.915) suggest that the first-order model adequately described the observed attenuation trends. The attenuation coefficient in the wet season is generally higher than that in the dry season, likely due to the stronger hydrodynamic conditions during the wet season that enhances the mixing and transformation of pollutants.

Antibiotics in rivers mainly undergo migration and transformation through multiple pathways such as hydrolysis, photodegradation, biodegradation, and sorption in sediments (Tran et al., 2015; Danner et al., 2019). The rapid decay of SCP may be related to the fact that its molecular structure is prone to photolysis and hydrolysis. Especially under conditions of high light and strong runoff during the wet season, these processes are more pronounced (Kulik et al., 2023). The result of this study is consistent with the existing literature’s’ conclusion that sulfonamide antibiotics are easily degradable (Chen et al., 2024; Ma et al., 2021). However, the *λ* value of SCP varied significantly among different river sections and seasons, reflecting the regulatory role of local hydrogeological and climatic conditions on the behavior of pollutants. Several uncertainties should be acknowledged. First, the estimated attenuation coefficients were associated with relatively wide 95% confidence intervals, reflecting the limited number of sampling points (*n* = 4 per regression). This limits the extent to which the assumed first-order attenuation behavior can be independently assessed. Second, while we selected transects without major tributaries or point sources, diffuse inputs from agricultural runoff cannot be entirely excluded, and our λ estimates may therefore represent upper-bound apparent attenuation rates that include dilution and dispersion. Therefore, the attenuation coefficients obtained from field monitoring data in this study provide a preliminary but valuable insight into the environmental fate of antibiotics and a helpful basis for designing future controlled degradation experiments.

### Ecological risk assessment

**Fig. 4:**
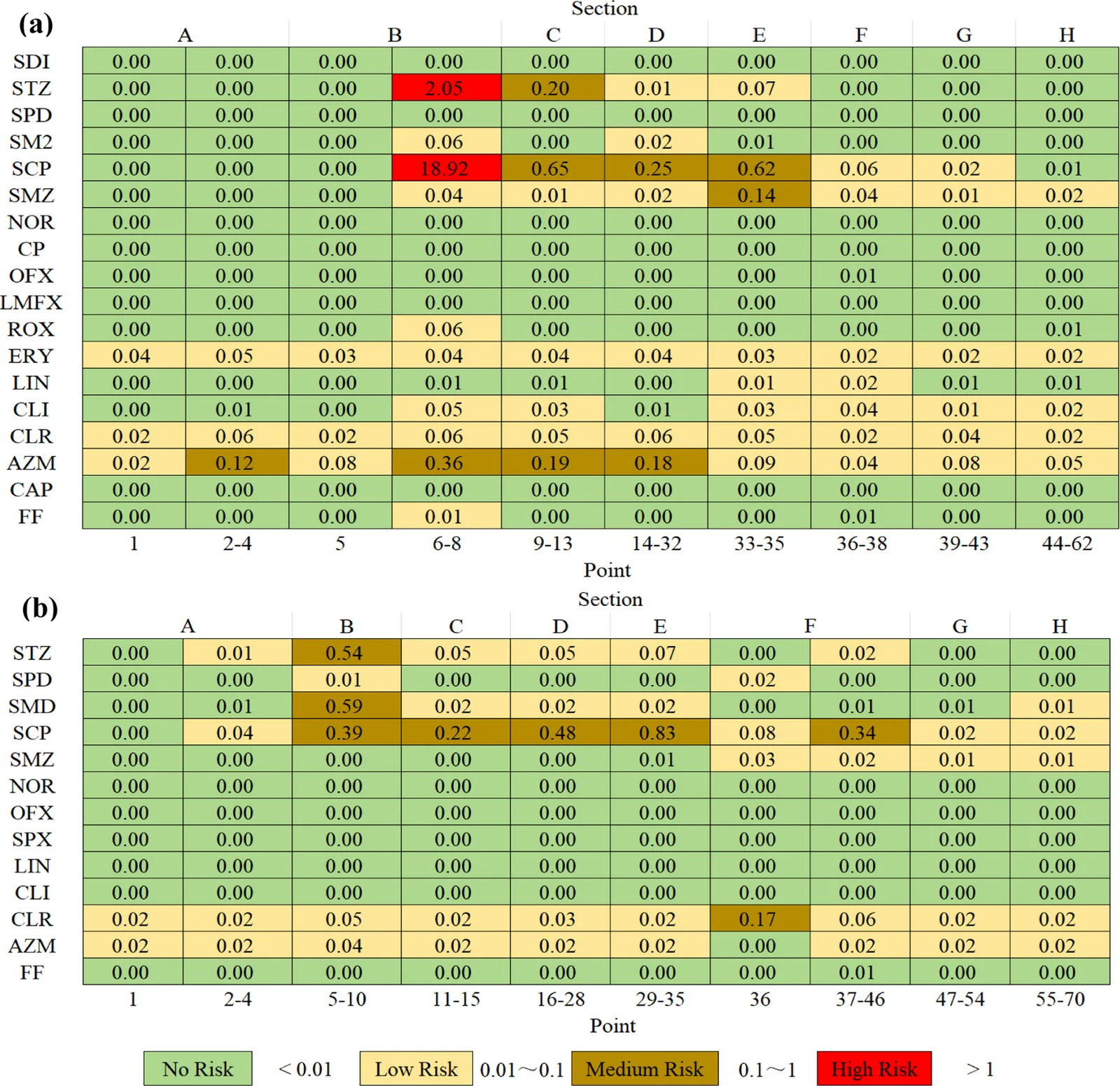
**a** Risk quotients (RQs) of the antibiotics at the sampling points of different river sections in 2024; **b** RQs of the antibiotics at the sampling points of different river sections in 2025. Undetected compounds are excluded. Abbreviations: AZM, azithromycin; CLR, clarithromycin; ERY, erythromycin; STZ, sulfathiazole; SCP, sulfachloropyridazine; SM2, sulfamethazine; ROX, roxithromycin; SMZ, sulfamethoxazole; CLI, clindamycin; FF, florfenicol; OFX, ofloxacin; CAP, chloramphenicol; SPD, sulfapyridine; LIN, lincomycin; SMD, sulfameter; SDI, sulfadiazine; NOR, norfloxacin; CP, ciprofloxacin; LMFX, lomefloxacin; OFX, ofloxacin; SPX, sparfloxacin

The RQ values of different antibiotics at various points are calculated to systematically assess the potential risks they pose to the aquatic ecosystem of the river basin. The assessment results reveal significant spatial heterogeneity and compound specificity. Figure 4 shows RQ values of the detected antibiotics in the river basin in 2024 and 2025, with those undetected (DFs = 0) excluded. Overall, most antibiotics were in a no risk state (RQ < 0.01) in most river sections, but there was a significant spatial heterogeneity and risk aggregation phenomenon. In 2024, SCP showed an extremely high-risk level at the sampling points 6–8 (average RQ = 18.92), significantly higher than that at all other locations, constituting the main source of risk. Additionally, antibiotics such as STZ and AZM exhibited a moderate to low-risk diffusion trend in multiple consecutive intervals (points 6–8, 9–13, 14–32, etc.). Among them, the mean RQ of STZ at points 6–8 reached 2.05, and the mean RQ of AZM at the 6–8, 9–13, and 14–32 intervals were 0.36, 0.19, and 0.18, respectively. It is worth noting that although the risk levels of ERY and other antibiotics were relatively low (0.02–0.06), there was still a continuous low-level persistent risk at all intervals.

In comparison, the risks in 2025 were mainly concentrated on three antibiotics: STZ, SMD, and SCP. Specifically, the mean RQs of STZ and SMD reached 0.54 and 0.59, respectively, at points 5–10, falling in the “moderate risk” category (0.1–1). SCP showed a more extensive and persistent risk, with its mean RQs being relatively high in the river sections B, D, and E (0.39, 0.48, 0.48, respectively), and also presenting “moderate risk” (0.34) at points 37–46. Additionally, CLR and AZM exhibited persistently “low risk” (0.01–0.1) at all points. Compared to 2024, the overall risk level in 2025 was more moderate, and no high-risk level points emerged. Although the high-risk characteristics of SCP and STZ in 2024 significantly decreased in 2025, they were detected as being in the medium-risk category in 2024. Especially for SCP, it was still detected to have medium risk in several river sections in 2024. This conclusively proves that SCP was a key risk indicator for the aquatic ecosystem in this river basin. STZ, AZM, CLR, and others consistently exhibited moderate to low risks at different sites across the 2 years, forming the background risk spectra of this river basin. The risks can be highly localized and must be identified through localized monitoring for their “characteristic risk pollutants” and “key risk source areas.”

Several limitations of this risk assessment should be acknowledged. First, the PNEC values were derived from the ECOSAR model, which may not fully reflect species-specific sensitivity and realistic environmental exposure conditions. Although the application of a fixed assessment factor (AF = 1000) provides a conservative screening approach, it also introduces uncertainty. Second, this study assessed the risks of individual antibiotics, while the potential mixture toxicity among co-occurring antibiotics (e.g., SCP–SMZ and STZ–AZM in Section B) was not considered. Therefore, the actual ecological risks may be underestimated due to combined effects among multiple antibiotics. Overall, the RQ results should be interpreted as a preliminary screening-level assessment for identifying priority pollutants and guiding further investigations into chronic toxicity and mixture effects.

### Prioritization framework

Based on a comprehensive analysis integrating DFs, concentration levels, and ecological RQs, a prioritized monitoring framework for antibiotics in this watershed is established. The 24 antibiotics, including 14 undetected and 10 with both low detection (< 20%) and risk (< 0.1) can be excluded from routine monitoring. However, because occurrence patterns may shift over time, we recommend a biennial or triennial reassessment, as well as event-triggered re-evaluation following significant land-use changes, to confirm their continued deprioritization. The remaining eight antibiotics warrant focused attention. Among them, SCP is identified as the core risk indicator due to its extremely high localized concentration and dominant ecological risk, forming a distinctive pollution fingerprint. Meanwhile, STZ and SMZ are also priority pollutants showing significant concentrations and medium risk in specific zones in 2024. Additionally, the other five antibiotics constitute a suite of background risk monitoring compounds, reflecting persistent and widespread low-to-medium level inputs. Moreover, the priority of compounds is not constant throughout the entire watershed but should be combined with specific land use types. For example, in agricultural areas dominated by livestock farming and crop cultivation, SCP should be given priority consideration.

Our framework integrates three orthogonal lines of evidence which collectively reduce the risk of false-positive or false-negative designation. Previous prioritization studies have applied multi-criteria decision-making approaches that combine exposure, ecological effects, and human health risk to generate a unified priority list for an entire watershed (e.g., Liu et al., 2025). However, they generally do not account for the spatial heterogeneity of pollution sources within a river system—a given antibiotic may pose high risk in one river section but remain negligible in another. Our framework addresses this limitation by being spatially explicit. The designation of SCP as a core indicator is specifically tied to agricultural hotspot (Section B), whereas SMZ and FF are prioritized in urban-influenced Section D, enabling zone-specific monitoring strategies rather than a uniform basin-wide list. Moreover, the framework explicitly identifies a substantial number of antibiotics (24 of 32) that can be excluded from routine monitoring based on consistent absence or negligible risk. Thus, a practical advantage can be directly translated to resource optimization for regulatory agencies. To our knowledge, this integrated, spatially adaptive, and attenuation-informed prioritization approach has not been previously applied to river basin-scale antibiotic monitoring.

## Conclusions

The study reveals significant spatiotemporal heterogeneity in antibiotic occurrence, with DFs of 0–82.86%, concentrations of ND–36,577 ng/L, and RQs of 0–18.92 in the river basin of eastern China. Based on these three parameters, SCP, STZ, and SMZ are recommended as priority indicators for cost-effectively tracing aquaculture-derived contamination, while 24 antibiotics can be deprioritized to optimize monitoring resources with periodic reassignment. Spatially, the distinct pollution patterns necessitate zone-specific interventions. At agricultural hotspots like the river section B, characterized by SCP-driven “high-intensity, rapid-attenuation” peaks, efforts should focus on enhancing livestock waste utilization and discharge control. In urban-influenced areas like the river section D, where pollutants such as SMZ and FF exhibit a “moderate-intensity, slow-decay” pattern, priority should be given to improving sewage networks and advanced treatment capabilities. These findings underscore the high environmental heterogeneity of antibiotic pollution and the need for adaptive management strategies. More broadly, this study provides a transferable framework integrating tiered monitoring with spatially adaptive management that can be applied to other watersheds facing similar pollution complexities. However, since our source identification is based on spatial and land use correlations rather than formal source apportionment, these management recommendations should be regarded as provisional. They can be further adjusted once quantitative source apportionment data is available.

Compared with previous river surveys, this study is the first to quantitatively estimate the attenuation coefficient (0.28–2.24 km⁻^1^) of SCP in a river in eastern China and also analyze its seasonal variations. These values should be interpreted as apparent field attenuation coefficients—integrating dilution, hydrodynamic mixing, sorption, and transformation—rather than intrinsic degradation constants, and their uncertainty (e.g., limited sampling points, potential diffuse inputs) warrants further validation through controlled experiments and higher-resolution longitudinal sampling. Furthermore, a comprehensive and evidence-based prioritization framework is established. This framework can reduce the list of target analytes by more than 70%, while maintaining the detection sensitivity for high-risk compounds. This practical outcome has direct significance for resource-constrained regulatory monitoring programs.

Future research should focus on elucidating the precise degradation pathways (photolysis, hydrolysis, microbial degradation) of SCP and other key antibiotics, as well as their interactions with sediments and dissolved organic matter under controlled conditions. Moreover, refined risk assessments using experimentally derived toxicity data for locally relevant aquatic species, combined with mixture toxicity models (e.g., concentration addition or independent action models), are needed to better represent the actual ecological risks posed by the complex antibiotic mixtures observed in this basin.

## Supplementary Information

Below is the link to the electronic supplementary material.

Supplementary file1 (DOCX 3.33 MB)

## Data Availability

The datasets generated and/or analyzed during the current study are available from the corresponding author on reasonable request.
