# Supplementary material for: Occurrence, spatiotemporal distribution, and ecological risks of antibiotics in a river basin of eastern China
Source: Environ Monit Assess. 2026 Oct 16;198(11):1204. doi: 10.1007/s10661-026-16036-z (PMC13672664; doi:10.1007/s10661-026-16036-z)
Supplement: Supplementary file 1 [file 10661_2026_16036_MOESM1_ESM.docx]

**Supporting Information:**

**Occurrence, Spatiotemporal Distribution, and Ecological Risks of Antibiotics in a River Basin of Eastern China**

Xintong Lin^a, *^, Jing Wang^b^, Zhengzheng Liu^b^, Jingqing Zhou^b^, Qinqin Sun^b^, Shuxin Chen^b^, Huayu Hu^b^

^a^ School of Civil and Environmental Engineering, The University of New South Wales, Sydney, NSW 2052, Australia

^b^ Ecological and Environmental Monitoring Center of Zhejiang Province, Hangzhou 310012, China

*Corresponding Author：E-mail: z5701054@ad.unsw.edu.au

**Table S1. CAS Number and Limit of Quantification of Target Antibiotic**

| Antibiotics | | Abbr. | CAS Number | Limit of quantification (LOQ)  (ng/L) |
| --- | --- | --- | --- | --- |
| sulfonamides  (SAs) | Sulfadiazine | SDI | 68-35-9 | 0.4 |
|  | Sulfathiazole | STZ | 72-14-0 | 0.4 |
|  | Sulfapyridine | SPD | 144-83-2 | 0.4 |
|  | Sulfamerazine | SM | 127-79-7 | 0.8 |
|  | Sulfamethazine | SM2 | 1981-58-4 | 0.4 |
|  | Sulfamonomethoxine | SMM | 1220-83-3 | 0.4 |
|  | Sulfamethizole | SMTZ | 144-82-1 | 0.8 |
|  | Sulfameter | SMD | 651-06-9 | 0.8 |
|  | Sulfachloropyridazine | SCP | 80-32-0 | 0.8 |
|  | Sulfamethoxazole | SMZ | 723-46-6 | 0.4 |
| fluoroquinolones  (FQs) | Enrofloxacin | ENR | 93106-60-6 | 4.0 |
|  | Norfloxacin | NOR | 70458-96-7 | 2.4 |
|  | Ciprofloxacin | CP | 85721-33-1 | 2.0 |
|  | Ofloxacin | OFX | 82419-36-1 | 2.0 |
|  | Sarafloxacin | SAR | 98105-99-8 | 2.0 |
|  | Enoxacin | ENX | 74011-58-8 | 2.0 |
|  | Lomefloxacin | LMFX | 98079-51-7 | 4.4 |
|  | Nalidixic acid | NA | 389-08-2 | 1.6 |
|  | Flumequine | FLU | 42835-25-6 | 0.8 |
|  | Cinoxacin | CIN | 28657-80-9 | 0.8 |
|  | Sparfloxacin | SPX | 110871-86-8 | 2.0 |
| tetracyclines  (TCs) | Oxytetracycline | OTC | 79-57-2 | 2.0 |
|  | Tetracycline | TC | 60-54-8 | 4.0 |
|  | Chlortetracycline | CTC | 57-62-5 | 4.0 |
| macrolides  (MCs) | Roxithromycin | ROX | 80214-83-1 | 0.4 |
|  | Erythromycin | ERY | 114-07-8 | 10.0 |
|  | Clarithromycin | CLR | 81103-11-9 | 3.6 |
|  | Azithromycin | AZM | 83905-01-5 | 3.6 |
| lincosamides  (LINs) | Lincomycin | LIN | 154-21-2 | 0.4 |
|  | Clindamycin | CLI | 21462-39-5 | 0.4 |
| amphenicols  (AMPs) | Chloramphenicol | CAP | 56-75-7 | 0.8 |
|  | Florfenicol | FF | 73231-34-2 | 0.4 |

Note: LOQ was determined as the lowest point on the calibration curve with a signal-to-noise ratio ≥10 and accuracy within 80-120%.

**Table S2. UPLC-MS/MS Instrumental Parameters: Gradient Program**

| **Time (min)** | **Liquid Phase A (%)** | **Liquid Phase B (%)** |
| --- | --- | --- |
| 0 | 90 | 10 |
| 1 | 90 | 10 |
| 6 | 70 | 30 |
| 7 | 30 | 70 |
| 9 | 15 | 85 |
| 10 | 15 | 85 |
| 10.1 | 90 | 10 |
| 12 | 90 | 10 |

Note: Mobile Phase A: 0.1% (v/v) formic acid in water; Mobile Phase B: methanol.

**Table S3. List of LC_50_ of the antibiotics.**

| Antibiotics | LC_50_ (mg/L) |
| --- | --- |
| SDI | 907 |
| STZ | 90 |
| SPD | 246 |
| SM2 | 62.1 |
| SMD | 899 |
| SCP | 391 |
| SMZ | 267 |
| NOR | 2010 |
| CP | 1310 |
| OFX | 1940 |
| LMFX | 8670 |
| ROX | 51.6 |
| ERY | 68.4 |
| LIN | 1040 |
| CLI | 80.8 |
| CLR | 24.2 |
| AZM | 21.9 |
| CAP | 2500 |
| FF | 1100 |
| SPX | 1240 |

Note: LC₅₀ values were primarily sourced from the U.S. EPA’s ECOSAR model (v2.0) or peer-reviewed ecotoxicity studies where available; Predicted No-Effect Concentration (PNEC) was calculated as: PNEC = LC₅₀ / Assessment Factor (AF). A conservative AF of 1000 was applied to extrapolate from acute laboratory data to chronic environmental protection, following standard guidelines.

**Figure S1. Map of Esri Land Cover land use type**

Note: The sampling points are identified by the labels from the sampling year 2024. The area within the rectangular frame is predominantly yellow (Crops) interspersed with red (Built Area), exhibiting a mosaic pattern of cropland and construction land, which is characteristic of an agricultural region. In contrast, the area within the circular frame is almost entirely red, indicating a densely built environment, which can be inferred as a contiguous residential zone or urban-influenced area.

**Figure S2.** **Detection frequencies (DFs) of the 32 antibiotics in 2024 and 2025.**
